# Modulating Metabolism to Improve Cancer-Induced Muscle Wasting

**DOI:** 10.1155/2018/7153610

**Published:** 2018-01-29

**Authors:** Fabio Penna, Riccardo Ballarò, Marc Beltrá, Serena De Lucia, Paola Costelli

**Affiliations:** ^1^Department of Clinical and Biological Sciences, University of Torino, Turin, Italy; ^2^Interuniversity Institute of Myology (IIM), Urbino, Italy

## Abstract

Muscle wasting is one of the main features of cancer cachexia, a multifactorial syndrome frequently occurring in oncologic patients. The onset of cachexia is associated with reduced tolerance and response to antineoplastic treatments, eventually leading to clinical conditions that are not compatible with survival. Among the mechanisms underlying cachexia, protein and energy dysmetabolism play a major role. In this regard, several potential treatments have been proposed, mainly on the basis of promising results obtained in preclinical models. However, at present, no treatment yet reached validation to be used in the clinical practice, although several drugs are currently tested in clinical trials for their ability to improve muscle metabolism in cancer patients. Along this line, the results obtained in both experimental and clinical studies clearly show that cachexia can be effectively approached by a multidirectional strategy targeting nutrition, inflammation, catabolism, and inactivity at the same time. In the present study, approaches aimed to modulate muscle metabolism in cachexia will be reviewed.

## 1. Introduction

Cancer-induced muscle wasting is one of the hallmarks of cachexia, a multifactorial syndrome that represents one of the most important comorbidities in oncologic patients. The occurrence of cachexia markedly complicates the management of cancer patients, negatively impinging on the tolerance and response to antineoplastic treatments, worsening the quality of life, and reducing survival. In particular, about 25% of deaths by cancer are due to cachexia, rather than to the tumor itself [1].

Few years ago, a classification of cachexia was proposed, defining three different stages: precachexia, cachexia, and refractory cachexia [2]. Prognosis progressively worsens going from patients with precachexia to those with refractory cachexia. In this regard, the earlier anticachexia treatments are set up, the better. For this reason, the research on cachexia is focused on two main goals: (i) to find out biomarkers useful to the early identification of a condition of still latent cachexia and (ii) to define treatment protocols useful to delay the progression from precachexia to refractory cachexia.

Skeletal muscle wasting in cancer patients has a good prognostic value, being predictor of reduced tolerance to chemotherapy and/or surgery, decreased ability to perform daily activities, and shortened survival. In addition, recent data report that loss of muscle mass negatively affects quality of life in cancer patients [3, 4]; such correlation might occur irrespectively of survival rates [5]. Being poor quality of life one of the most prominent and invalidating consequences of cancer cachexia, to investigate the mechanisms underlying cancer-induced muscle wasting is even more relevant to design therapeutic strategies that also take into account patient well-being.

The possibility to underestimate the occurrence of muscle mass depletion exists, since the first approach to clinically evaluate a patient is to obtain information about body weight and body mass index (BMI). However, in face of no body weight loss and/or normal BMI, reduced muscle mass might well occur, being masked by fat or water content. Another relevant point that frequently is poorly taken into consideration is that the loss of muscle mass is likely exacerbated by anticancer treatments.

At present, different mechanisms have been proposed to lead to muscle wasting in cancer hosts, among which there are altered protein and energy metabolism and impaired myogenesis [1]. Several factors may contribute to these alterations, such as reduced calorie intake, hormonal unbalance, and systemic inflammation.

Cancer-driven production of proinflammatory cytokines plays a relevant role in tumor progression and markedly contributes to cachexia. Indeed, in cancer patients, systemic inflammation correlates with increased resting energy expenditure and with reduced survival rate [6]. Along the same line, increased circulating levels of tumor necrosis factor *α* (TNF*α*), interleukin-6 (IL-6), *γ*-interferon (INF), and, more recently, growth and differentiation factor 15 (GDF15) have been reported in cachectic cancer patients [1, 7]. The link existing between cytokines and cachexia has led to the inclusion of anti-inflammatory drugs in treatment protocols [8].

The present review will focus on strategies able to modulate metabolism that may reveal useful to prevent/delay cancer-induced muscle wasting.

## 2. Protein and Amino Acid Metabolism

Altered protein turnover is a general feature of muscle wasting in cancer cachexia. In particular, protein breakdown rates are persistently increased, while protein synthesis rates can be reduced, unchanged, or even increased, depending on the model system [1]. The different reaction kinetics that characterizes protein synthesis and degradation rates, zero and first order, respectively, implies that if degradation is higher than normal, the loss of total protein cannot be antagonized by simply increasing the rate of synthesis. Taking this assumption for true, any anabolic approach should be associated with anticatabolic strategies in order to achieve a beneficial effect on muscle protein mass.

### 2.1. Protein Degradation

**Figure 1:**
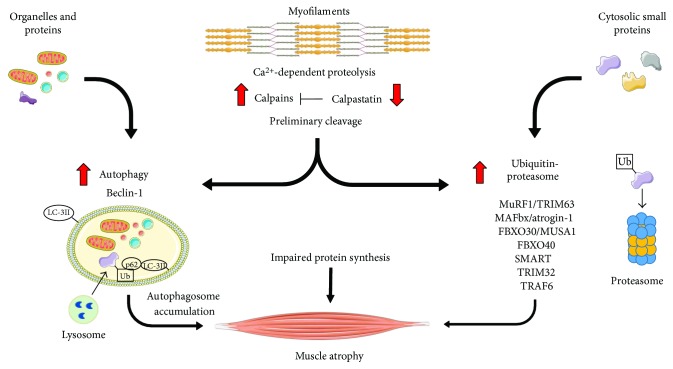
Main catabolic pathways contributing to protein breakdown in cancer cachexia.

Several pieces of evidence suggest that the intracellular proteolytic systems in the skeletal muscle of cancer hosts are poised towards activation above the physiological levels (Figure 1). Particularly relevant in this regard are the pathways dependent on ubiquitin-proteasome and autophagy. While the former mainly degrades short-lived and regulatory proteins, the latter gets rid of structural proteins and organelles [9].

Both experimental and human cancer cachexia were associated with increased activity of the ubiquitin-proteasome pathway [1]. Of interest, alterations in molecular and biochemical markers of proteasome activation were observed in gastric cancer patients before any evidence of body weight loss, supporting the need to detect cachexia as early as possible [10]. Modulations of the ubiquitin-proteasome proteolytic system, however, are not a general finding in cancer cachexia, as shown by studies reporting that it is not differently activated with respect to controls in the muscle of patients affected by non-small-cell lung cancer (NSCLC; [11]) or esophageal cancer [12].

The involvement of the autophagic-lysosomal proteolysis in muscle wasting was recognized just in the last fifteen years. Two main reasons account for such delay: (i) autophagy was not considered as typically operated by the skeletal muscle as a response to stress conditions. Such belief was definitely abandoned when autophagy was clearly demonstrated in fasted mice overexpressing green fluorescent protein-labeled LC3 [13]. (ii) The study and detection of autophagy was not easy since the ATG genes were not discovered [14]. A number of studies reported that the autophagic system was overactivated, without reaching complete cargo degradation, in the muscle of both tumor-bearing animals and cancer patients [6, 15–17]. In particular, despite autophagic flux was enhanced in mice bearing the C26 tumor, autophagosomes accumulated, likely due to exhaustion of the lysosomal compartment [15]. A similar pattern could also be observed in cancer patients, as suggested by LC3B-II and p62 accumulation [16, 17].

Both proteasome and lysosomes, however, cannot directly degrade intact myofilaments. In this regard, a preliminary cleavage was proposed to be operated by other proteolytic systems, such as those dependent on caspases or calpains. These latter are Ca^2+^-dependent cysteine proteases, normally inactive and localized in the cytosolic compartment. When intracellular Ca^2+^ concentrations increase, inactive calpains translocate to the cell membrane and become activated by autoproteolysis [18]. The system also includes calpastatin, a physiological inhibitor, which is a substrate of active calpain itself. Increased calpain expression was reported in the muscle of tumor-bearing animals [19], while rats transplanted with the Yoshida AH-130 hepatoma showed a progressive reduction of calpastatin levels and increased *in vitro* cleavage of specific fluorogenic substrates [20]. More recently, calpain activation was also demonstrated in mice bearing the C26 colon carcinoma [21]. Both increased or unchanged muscle calpain expression were reported in cancer patients [12, 22].

Several lines of evidence show that proinflammatory cytokines act as triggers, or at least as contributors, of cancer-induced protein hypercatabolism [23]. Briefly, data obtained in both experimental models and human pathology have demonstrated that cytokines such as TNF*α* and IL-6 lead to reduced rates of protein synthesis paralleled by enhanced protein breakdown, both accounting for the loss of muscle mass [24]. Such effects depend, at least in part, on activation of the transcription factor NF-*κ*B, as shown in both experimental and human cancer cachexia patients [25, 26]. Cancer-induced muscle wasting has also been associated with another proinflammatory cytokine, namely, TNF-like weak inducer of apoptosis (TWEAK) [27].

The therapeutic approaches mainly pursued by researchers to counteract enhanced muscle protein breakdown have long been those specifically targeting the different proteolytic systems. The results, however, did not really clarify the issue.

Since the discovery of muscle-specific ubiquitin ligases, these were considered a good target to interfere with protein breakdown, being involved in determining both substrate-specificity and proteasome degradation rate. Among the enzymes belonging to this family, the first described were MAFbx/atrogin-1 and MuRF1/TRIM63, respectively, involved in the degradation of structural proteins and of proteins contributing to cell proliferation, differentiation, and survival [1]. Subsequently, other members came out, such as TRIM32 and FBXO40. More recently, FBXO30/MUSA1 was shown to contribute to denervation- and fasting-mediated muscle loss [28], as well as to cancer-induced muscle wasting (unpublished data). Genetic approaches specifically targeting these ubiquitin ligases proved effective in protecting the muscle against protein depletion [29]; however, at present, the use of these enzymes as therapeutic targets for muscle wasting is not validated yet.

On the other side, the inhibition of proteasome activity by means of pharmacological inhibitors was effective just in few models of muscle atrophy but totally unable to protect tumor-bearing animals against muscle wasting [30]. In contrast with these findings, few years ago, a study reported that inhibition of the ubiquitin-proteasome pathway by MG132 was able to improve experimental cancer cachexia [31]. However, MG132 is a rather unspecific inhibitor, being able to block also calpains and autophagy [31, 32]. Finally, carfilzomib, an irreversible selective inhibitor of proteasome chymotrypsin-like activity, was shown to improve cachexia in tumor-bearing mice by inhibiting muscle protein breakdown [31]. Such improvement, however, was associated with reduced tumor burden, which could be the real mechanism underlying the beneficial effect of the treatment.

Several lines of evidence proposed that modulations of autophagy could be useful to improve cancer-associated muscle wasting. In this regard, muscle-specific gene strategies aimed at silencing Beclin-1, one of the proteins involved in autophagosome formation, showed that suppression of autophagy in the C26 hosts was unable to rescue myofiber diameter (unpublished data). In addition, pharmacological inhibition of autophagy in mice hosting the C26 tumor lead to death of the animals, suggesting that lysosomal degradation is mandatory to sustain the requirement of both energy and substrates in tumor hosts, at least when they are facing the terminal phase of cancer growth [15]. The other way round, excessive stimulation of muscle autophagy, experimentally obtained by the overexpression of TP53INP2/DOR, exacerbated muscle atrophy in tumor-bearing mice (unpublished data), while activation of autophagy by means of the mTOR inhibitor rapamycin was shown to positively affect the skeletal muscle in the C26 hosts [16]. Such discrepancy might depend on the fact that while mTOR inhibition affects stress-induced autophagy, TP53INP2 hyperexpression targets basal autophagy.

Despite the literature report data supporting the involvement of Ca^2+^-dependent proteolysis in the pathogenesis of cancer-induced muscle wasting, protein hypercatabolism was not downregulated in preparations of isolated muscles obtained from tumor-bearing animals and incubated in the presence of calpain inhibitors [19, 33, 34]. More recently, both pharmacological and genetic approaches aimed at inhibiting the Ca^2+^-dependent proteolytic system were not able to prevent or delay cancer-induced muscle wasting [21], although contrasting results were reported in this regard [35].

While interfering with specific proteolytic systems does not seem to be an appropriate approach to prevent/delay cancer-induced muscle wasting, the modulation of bulk protein turnover appears more promising. In this regard, anti-inflammatory approaches revealed able to improve muscle protein turnover in tumor-bearing mice [20]. More recently, administration of formoterol, a *β*_2_-adrenergic agonist, to tumor-bearing animals revealed able to reverse muscle wasting [36]. Such an effect is mainly exerted by stimulating protein synthesis and inhibiting protein degradation rates. In particular, both the ubiquitin-proteasome and the autophagic-lysosomal proteolytic systems were downregulated in formoterol-receiving animals ([24]; unpublished data). At present, only one study evaluated the effectiveness of formoterol, combined with megestrol acetate, in cachectic cancer patients [37]. The results suggest that both muscle size and strength can be improved by the treatment, although more trials are needed to draw clear-cut evidence.

### 2.2. Protein Synthesis

As reported above, depending on the situation, reduced, normal, or even increased muscle protein synthesis rates were shown in cancer cachexia. Due to the rapid development of cachexia, tumor-bearing animals frequently show reduced protein synthesis rates, although this is not a general finding. Indeed, rats bearing the AH-130 hepatoma, that usually die about 10 days after tumor transplantation, showed muscle protein synthesis rates comparable to those of healthy animals [38]. The situation is more complex when studying human pathology. Reduced muscle protein synthesis was reported many years ago in patients affected by different types of tumors [39] and more recently in prostate cancer patients [40]. On the contrary, van Dijk et al. [41] reported that baseline protein synthesis rates were higher than control values in cachectic patients affected by pancreatic cancer. Also, intermediate results are available in the literature: myofibrillar protein synthesis rates were analyzed in healthy people and weight-stable and weight-losing gastrointestinal cancer patients and found comparable among the different groups. Similarly, no changes in whole body protein synthesis were reported in NSCLC patients [42].

The possibility to modulate protein synthesis in order to correct muscle atrophy or simply to provide an environment permissive for the maintenance of muscle mass was long studied. Many approaches were tested, most of them consisting in nutritional strategies or in molecular modulations aimed at pushing muscle metabolism towards anabolism. Most of these approaches revealed unsuccessful, giving rise to the idea that anabolic resistance occurs in cancer cachexia. Just to provide few examples, conventional nutritional supplementation or the infusion with an amino acid cocktail did not stimulate muscle protein synthesis in advanced cancer patients [42]. Along this line, studies aimed at stimulating the anabolic pathway depending on IGF-1, by both pharmacological and genetic means, did not succeed in improving muscle wasting in tumor-bearing animals [43, 44].

Recently, however, patients not yet considered as refractory cachectic were proposed to display an anabolic window that could be exploited with nutritional interventions [42, 45, 46] or with other anabolism-inducing strategies. As an example, patients with stage III and IV NSCLC showed a normal anabolic response to hyperaminoacidemia but not to isoaminoacidemia, suggesting that high substrate availability is relevant to induce anabolism in cancer hosts [47]. Consistently, muscle protein synthesis could be stimulated in advanced cancer patients by a high protein formula versus a conventional nutritional supplement [48, 49]. These observations point out the possibility to overcome the anabolic resistance that occurs in cancer patients by providing specifically enriched nutritional supplements.

Stimulation of anabolism can be exerted by several means. Particularly interesting in this regard is ghrelin, a mediator released by the stomach during fasting or caloric restriction. Modulations of ghrelin levels exert remarkable effects on both energy and protein metabolism, such as the inhibition of autophagy in conditions characterized by systemic inflammation [50]. The administration of ghrelin to tumor-bearing animals improved food intake, body weight, lean body mass, and chemotherapy-induced toxicity [51]. Both ghrelin and ghrelin analogues are currently being tested in clinical trials. Among these, anamorelin was recently shown to improve lean body mass, total body mass, and hand grip strength in patients affected by NSCLC [52]. Other studies, however, showed that anamorelin administration to cancer patients increased body weight and improved FAACT scores while did not enhance hand grip strength [53].

### 2.3. Amino Acids

In addition to be necessary to synthesize proteins, free amino acids (FAA) also act as regulators of protein metabolism. In particular, plasma FAA, that even represent a small fraction of the total amino acid pool, are the main source of metabolically active nitrogen compounds. Among FAA, the essential amino acids were reported to stimulate protein synthesis and to inhibit protein degradation. Such a role is mainly played by the three branched chain amino acids (BCAA), leucine in particular.

Alterations of amino acid metabolism are frequent features in cancer-induced muscle wasting. Reduced amino acid uptake was generally observed in cancer patients, mainly due to the occurrence of anorexia, which also leads to decreased insulin secretion. Both decreased amino acid availability and insulin levels inhibit the anabolic pathway dependent on mTOR, resulting in downregulation of protein synthesis rates and stimulating protein degradation. Inhibition of mTOR signaling in cancer cachexia is enforced by proinflammatory cytokines [1]. Reduced amino acid uptake in the muscle was also reported to derive from altered amino acid transport. Indeed, in the soleus muscle of tumor-bearing rats, the activity of system A was decreased, while no changes were observed for systems L and ASC [54]. Of interest, TNF*α* was shown to impair amino acid transport in tumor-bearing rats [55]. Plasma glutamine levels were shown to be significantly reduced in tumor-bearing rats with respect to healthy animals [56]. Of interest, reduced glutamine availability could activate the metabolic sensor adenosine monophosphate-activated protein kinase (AMPK, see below; [57]). Finally, leucine oxidation was markedly increased in the muscle of rats bearing the Yoshida AH-130 hepatoma [58]. Consistently, enhanced activity of the BCAA dehydrogenase was reported in rats bearing the Walker 256 carcinoma [59].

Several studies have proposed amino acid supplementation as a mean to improve cancer-induced muscle wasting. In experimental models of cancer cachexia, BCAA were shown to attenuate the loss of muscle mass. The underlying mechanisms of such effect are not clear, although downregulation of protein degradation and stimulation of protein synthesis were hypothesized [60]. More recently, metabolomic alterations were proposed to be the basis of the positive effects exerted by a leucine-rich diet on cachexia in rats bearing the Walker 256 carcinoma, in the absence of effects on tumor mass [61]. As for clinical studies, BCAA were proposed to improve anorexia [62], thus removing, partially at least, one of the mechanisms accounting for reduced amino acid uptake. Other studies supported a beneficial role of BCAAs on muscle protein metabolism, although this should be confirmed by larger randomized, blind, placebo-controlled trials [63].

Beta-hydroxy-beta-methylbutyrate (HMB), a metabolite of leucine, was shown to improve muscle wasting in experimental cancer cachexia, mainly by inhibiting protein degradation rather than stimulating protein synthesis [63]. Recently, HMB was proposed to be more effective than leucine in preventing body weight loss in tumor-bearing animals [64]. Such effect, however, could depend on the model system chosen, since HMB does not appear able to modulate muscle mass in mice bearing the C26 tumor (Costelli et al., unpublished observations). The situation is even more confused in human cachexia. Increase of both hemoglobin levels and fat-free mass were reported in advanced cancer patients administered a nutritional supplement containing HMB, arginine, and glutamine [65, 66]. Another study, however, was not able to demonstrate a beneficial effect of the same supplement in cancer patients [67], suggesting that the effectiveness of HMB in the clinical practice is still unclear and deserves further investigation.

Glutamine supplementation was reported to attenuate muscle protein wasting in cancer patients [68], as well as to improve the energy balance in rats bearing the Walker 256 tumor [69]. Finally, promising data are available about the possibility to treat cancer hosts with L-carnitine, an amino acid derivative that plays a role in fatty acid metabolism and energy production [63].

## 3. Energy Metabolism

A negative energy balance, generally resulting from both reduced production and increased expenditure, is a frequent occurrence in cancer patients. While resting energy expenditure (REE) frequently increases, likely due to enhanced thermogenesis, the occurrence of reduced physical activity, particularly in advanced cancer patients, paradoxically leads to a net decrease of total energy expenditure. The increased thermogenesis is consistent with the observation that in cachectic tumor-bearing animals, the expression of the brown adipose tissue- (BAT-) specific uncoupling protein 1 (UCP1) is higher than in controls, while UCP2 (ubiquitous) and UCP3 (expressed in BAT and muscle) levels increase in the skeletal muscle only [70]. Similarly, muscle UCP3 mRNA levels were higher in weight-losing than in non-weight-losing cancer patients or controls [71].

The increase of REE in cancer cachexia is not a new observation; however, just recently, the underlying mechanisms start to be clarified. A central point in this regard is played by muscle mitochondria compartment, which is markedly affected in tumor hosts. Indeed, both morphological [72, 73] and functional alterations [74, 75] were reported in experimental tumor-bearing animals. In particular, mitochondrial uncoupling and reduced oxidative capacity were associated with myofiber shift from oxidative to glycolytic fibers [73]. Impairment of the mitochondrial compartment results in decreased ATP production, leading to an energy deficit that becomes even worse since it is coupled with steadily increased REE. Consistently, reduced ATP levels and increased activity of the energy sensor AMPK were shown in the muscle of tumor-bearing animals [72, 73]. The lack of an appropriate energy supply results in compromised cell function and reduced contractile force generation, leading to loss of muscle mass and strength.

**Figure 2:**
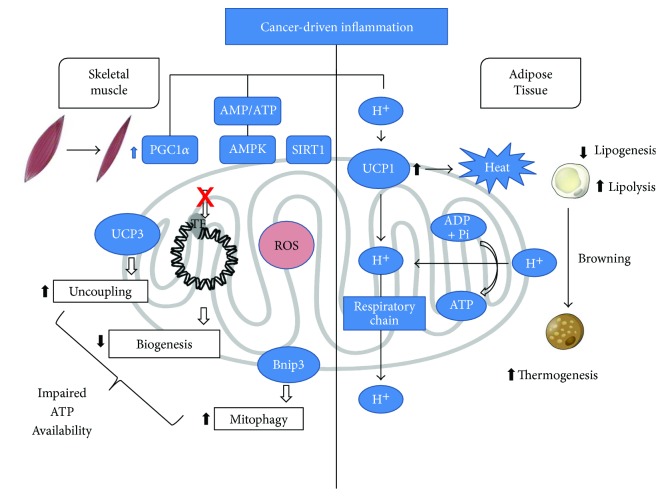
Mechanisms by which inflammation can impinge on muscle energy metabolism.

Several factors can lead to mitochondrial alterations in the skeletal muscle. Among these, proinflammatory cytokines play a major role. Indeed, the activation of NF-*κ*B induced by TNF*α* was reported to reduce both muscle oxidative capacity and the expression of factors regulating mitochondrial biogenesis. Similar observations were reported when other inflammation-driven pathways such as the IL-6/STAT3 or TGF*β*/Smad3 are activated above physiological levels [76]. In addition to proinflammatory mediators, also oxidative stress, due to reactive oxygen and nitrogen species levels exceeding the compensative capacity of the intracellular antioxidant systems, contributes to mitochondrial function impairment. In this regard, there are several studies sustaining the involvement of oxidative stress in cancer-induced muscle wasting, although a clear-cut causative evidence is still lacking [77] (Ballarò et al., unpublished data; Figure 2).

Being mitochondria crucial to the maintenance of muscle oxidative metabolism, emergency routes can be activated in order to avoid mitochondrial dysfunction. In particular, mitochondrial biogenesis and dynamics as well as the disposal of damaged organelles, mainly by mitophagy, are promoted (Figure 2). The other way round, impaired function of the emergency routes themselves could trigger the accumulation of altered mitochondria resulting in reduced muscle oxidative metabolism. In this regard, the expression of the peroxisome proliferator-activated receptor-*γ* (PPAR-*γ*) coactivator-1*α* (PGC-1*α*), the master regulator of mitochondrial biogenesis and oxidative metabolism, was shown to be reduced in the skeletal muscle of tumor-bearing mice [78], although this is not a constant finding [73, 79]. Mitochondria dynamics, representing the balance between fission and fusion processes, was shown to be altered in both experimental cancer cachexia and in cancer patients [78, 80]. In this regard, impaired mitochondrial dynamics could drive the hyperactivation of muscle protein breakdown, likely through pathways depending on AMPK and FoxO, eventually leading to muscle wasting [81, 82].

Autophagy (mitophagy) is the main mechanism responsible for disposal of altered mitochondria. Also, mitophagy was reported to be impaired in cancer cachexia, as shown by the observation that Bnip3L and Parkin1 mRNA increased in the muscle of cancer patients [17]. Similarly, Bnip3L protein levels were increased in the muscle of mice bearing the Lewis lung carcinoma [73]. On the whole, these observations suggest that in addition to mitochondria biogenesis and dynamics, also their disposal is perturbed in the skeletal muscle of tumor hosts, thus contributing to mitochondrial dysfunction and reduced muscle oxidative metabolism.

Several strategies were proposed to improve energy metabolism by acting on mitochondria. The first and perhaps simplest one, in theory at least, is exercise training, in particular a combination of both resistance and endurance exercise. These two types of training affect different but complementary targets, being able to improve force production and metabolic adaptations, respectively. Of particular relevance is the observation that endurance training was reported to increase the number of mitochondria and to drive myofiber-type shift from glycolytic to oxidative, thus specifically targeting alterations that characteristically occur in the skeletal muscle of tumor hosts. However, these potentially favorable effects can be exploited just systematically practicing exercise, even if at a moderate level. This may not be an easy task in cancer patients that frequently present with chronic fatigue and comorbidities eventually leading to exercise intolerance. This point is supported by the observation that C26-bearing mice did not benefit from exercise training, suggesting that the effort to exercise in already compromised animals was damaging rather than protective [73]. Consistently, excessive endurance exercise was associated with increased mitochondrial fission in the absence of mitophagy induction [83].

In the last years, the possibility to mimic the effects of exercise by drugs has been gaining a growing consensus. The positive side is that this strategy will allow to overcome both poor patient compliance to exercise training and possible occurrence of exercise intolerance. The negative part is that generally exercise mimicking drugs do not totally recapitulate the effects of exercise itself. In this sense, these drugs do not properly hit the target; however, they could be a good compromise when exercise training cannot be proposed to the patient.

At present, several options were investigated as exercise-mimicking strategies. While most of them are pharmacological, also a genetic approach was proposed. This latter consists in manipulations able to increase the levels of PGC-1*α* in the skeletal muscle. In this regard, improved exercise capacity and oxidative metabolism were reported in mice specifically overexpressing this factor in the muscle, resembling the phenotype induced by endurance training [84]. PGC-1*α* overexpression was shown to interfere with muscle atrophy induced by activation of the TWEAK-Fn14 pathway [85] and to improve cancer-induced muscle wasting in tumor-bearing mice [73, 86], although contrasting data were previously reported [87].

Several classes of drugs were proposed to modulate energy metabolism, among which are activators of AMPK, sirtuin 1 (SIRT1), and trimetazidine (TMZ).

Different compounds such as resveratrol, metformin, quercetin, and AICAR can activate AMPK [88]. In this regard, AICAR administration was shown to impinge on exercise capacity, oxygen consumption, and fatty acid oxidation [89]. Muscle atrophy induced by angiotensin II was prevented by treatment with AICAR. This drug also revealed able to activate autophagy and to improve muscle phenotype in both dystrophic *mdx* mice and animals bearing the C26 tumor [16, 90]. Metformin administration was shown to improve sarcopenia of aging and muscle wasting in severely burned patients [91, 92] and was proposed to be useful to treat muscle wasting in cancer cachexia [93]. AMPK activation can also be induced by resveratrol, as demonstrated by the observation that AMPK*α*_1_- or AMPK*α*_2_-deficient mice were refractory to resveratrol-induced increase of both mitochondrial biogenesis and endurance exercise capacity [94]. Consistently, obese men receiving resveratrol showed improved inflammation, AMPK activation, and increased expression of PGC-1*α* and SIRT1 protein levels [95]. Finally, an AMPK-stabilizing peptide was reported to improve white adipose tissue wasting in tumor hosts [96].

SIRT1 belongs to a class of deacetylases deregulated in aging and in different chronic diseases, including cancer. SIRT1 is also involved in the regulation of energy homeostasis; its expression is induced in response to caloric restriction [97] and can be activated in the skeletal muscle by AMPK [98]. Specific overexpression of SIRT1 in the muscle resulted in a fast-to-slow myofiber type transition, producing an oxidative phenotype. Consistently, muscle-specific SIRT1 transgenic mice exposed to fasting or denervation showed a reduced expression of atrogenes in comparison with wild-type littermates [99]. Finally, improved muscle phenotype was reported in *mdx*/SIRT1 double transgenic mice [100]. In addition to AMPK, resveratrol also activates SIRT1. In this regard, part of the above-described effects exerted by resveratrol derive from SIRT1-dependent modulations of PGC-1*α* acetylation state [101]. Synthetic selective SIRT1 activators such as SRT2104 are also available [102, 103]. Plasma lipid profile and insulin sensitivity were improved in healthy volunteers by administration of SRT2104 [102, 104]. Very few studies are actually available about SRT2104 action on both muscle mass and function. In this regard, SRT2104 appeared to reduce the depletion of muscle mass due to fasting or inactivity [105], at least in part by increasing PGC-1*α* expression [105].

TMZ is a metabolic modulator that blocks fatty acid oxidation, shifting ATP production to glucose oxidation and improving cell energy metabolism. Indeed, ATP synthesis through fatty acid *β*-oxidation requires more oxygen than glucose oxidation [106]. Along this line, the shift to glucose oxidation improves the use of the available oxygen, possibly increasing metabolic efficiency and skeletal muscle function. TMZ was shown to increase the size of cultured myotubes [107] and to improve both heart metabolism and exercise capacity in patients suffering from chronic stable angina [108]. When administered to aged animals, TMZ resulted in increased muscle strength [109]. Finally, treatment of C26-bearing mice with TMZ resembled some of the benefits triggered by exercise, among which fast-to-slow myofiber phenotype shift, PGC-1*α* upregulation, oxidative metabolism enhancement, and grip strength increase (Molinari et al., unpublished). The relevance of fatty acid oxidation to cachexia is also supported by a recent study showing that several tumor cell lines are able to release proinflammatory mediators, resulting in enhanced fatty acid oxidation and activation of p38-dependent signaling in the skeletal muscle, well before tissue wasting occurs. In addition, the same study also showed that treatment of tumor-bearing animals with etomoxir, an inhibitor of fatty acid oxidation, rescued both muscle mass and body weight [110].

## 4. Concluding Remarks

A complex network of metabolic alterations sustained by hypercatabolism, energy deficit, and systemic inflammation is the milieu underlying cancer cachexia. While becoming overtly detectable in advanced cancer patients, such perturbations likely take place very early in the course of the disease, at least at the molecular level.

Protein and energy dysmetabolism in cachexia are quite well recognized; however, the available therapeutic strategies, although frequently promising from the preclinical point of view, have not yet reached validation to be used in the clinical practice. Several drugs identified by experimental studies are currently tested in clinical trials for their ability to improve muscle metabolism in cancer patients. Other emerging strategies are those aimed at interfering with the intestinal microbiota, previously reported to improve cachexia in a preclinical model [111].

The available results of both experimental and clinical studies, however, have clearly indicated that single-targeted therapies will hardly be successful in the treatment of cachexia. In this regard, the view of a multidirectional approach, selectively tailored and, whenever possible, personalized, is gaining a growing consensus. Such an approach should not just rely on nutritional counseling and pharmacologic treatment with anti-inflammatory and anticatabolic drugs but also include exercise training and/or exercise-mimicking agents. In this regard, exercise mimetics could not only merely replace exercise training in depleted patients but also improve exercise tolerance and effectiveness in precachectic individuals, thus amplifying the beneficial action of exercise itself. Last but not least, treatments aimed at preventing/correcting the metabolic alterations underlying cancer-induced muscle wasting might also impinge on tumor-targeted therapies improving their effectiveness and/or enhancing patient tolerance to chemotherapy. In addition, metabolic modulators could also directly affect tumor growth. This is the case, for example, of exercise, that was shown to prevent or at least delay tumor growth [112].
